# *Edwardsiella tarda* Hfq: impact on host infection and global protein expression

**DOI:** 10.1186/1297-9716-45-23

**Published:** 2014-02-25

**Authors:** Yong-hua Hu, Yong-xin Li, Li Sun

**Affiliations:** 1 Key Laboratory of Experimental Marine Biology, Institute of Oceanology, Chinese Academy of Sciences, Qingdao 266071, China; 2 Taishan Vocational College of Nursing, 8 Ying Sheng East Road, Tai’an 271000, China

## Abstract

Hfq is an RNA-binding protein that plays an important role in many cellular processes. In this study, we examined the biological effect of the Hfq of *Edwardsiella tarda*, a severe fish pathogen with a broad host range that includes humans. To facilitate the study, a markerless *hfq* in-frame deletion wild type, TXhfq, was constructed. Compared to the wild type TX01, TXhfq exhibited (i) retarded planktonic and biofilm growth, (ii) decreased resistance against oxidative stress, (iii) attenuated overall virulence and tissue dissemination and colonization capacity, (iv) impaired ability to replicate in host macrophages and to block host immune response. Introduction of a *trans*-expressed *hfq* gene into TXhfq restored the lost virulence of TXhfq. To identify potential Hfq targets, comparative global proteomic analysis was conducted, which revealed that 20 proteins belonging to different functional categories were differentially expressed in TXhfq and TX01. Quantitative real time RT-PCR analysis showed that the mRNA levels of two thirds of the genes of the identified proteins were consistent with the proteomic results. Since TXhfq is dramatically attenuated in virulence, we further examined its potential as a naturally delivered vaccine administered via the immersion route in a flounder model. The results showed that TXhfq induced effective protection against lethal *E. tarda* challenge*.* Taken together, our study indicated that Hfq is required for the normal operation of *E. tarda* in multiple aspects, and that Hfq probably exerts a regulatory effect on a wide range of target genes at both transcription and post-transcription levels.

## Introduction

*Edwardsiella tarda* is a Gram-negative, motile, rod-shaped bacterium belonging to the family of *Enterobacteriaceae*. It is a serious fish pathogen that causes edwardsiellosis, a systematic disease that affects a wide range of farmed fish species including Japanese eel (*Anguilla japonica*, Temminck & Schlegel, 1847), Japanese flounder (*Paralichthys olivaceus*, Temminck & Schlegel 1846), turbot (*Scophtalmus maximus*, Linnaeus 1758), red sea bream (*Pagrus major*, Temminck & Schlegel 1843), tilapia (*Oreochromis niloticus*, Linnaeus 1758), and channel catfish (*Ictalurus punctatus*, Rafinesque 1818) [1]. Heavy economic losses due to *E. tarda*-related edwardsiellosis have been reported in aquaculture industries worldwide [2]. In addition to fish, *E. tarda* is also a human pathogen and known to cause gastroenteritis and extraintestinal infections [3]. Recent studies indicate that *E. tarda* possesses numerous virulence factors, which participate in different aspects of host infection [2,4]. Currently, owing to the lack of effective vaccines, control of *E. tarda* in aquaculture depends mainly on the use of antibiotics in most countries including China.

Hfq was originally identified as a host factor required for RNA replication of phage Qβ in *Escherichia coli*[5], and was classified into the conserved RNA-binding Lsm (like-Sm)/Sm-like protein family found in both eukaryotes and prokaryotes [6]. Accumulating evidences have shown that Hfq is an RNA chaperone involved in posttranscriptional gene regulation via several mechanisms, including interaction with small RNAs (sRNAs) and facilitating their binding to target mRNA, modulating mRNA degradation, and regulating the process of mRNA translation [7]. For pathogenic bacteria such as *Brucella abortus*, *E. coli*, *Legionella pneumophila*, *Pseudomonas aeruginosa*, *Salmonella* Typhimurium, *Vibrio cholerae*, *Yersinia enterocolitica*, and *Klebsiella pneumoniae*, Hfq is known to be a virulence factor [8-18]. Inactivation of the *hfq* gene leads to defect in a variety of biological aspects, notably cellular growth and motility, quorum sensing, stress tolerance, and infectivity [19,20]. However, to date very little study has been documented on the Hfq of fish pathogens, and for *E. tarda*, the role of Hfq is entirely unknown.

In the present study, we aimed to investigate the biological property of Hfq in *E. tarda* and, in particular, whether Hfq plays any role in *E. tarda* infection. For this purpose, we compared the effect of an *hfq* wild type, TXhfq, to that of the wild type strain. We found that deletion of *hfq* in *E. tarda* had pleiotropic effects on bacterial growth, infection, and global protein expression. Additionally, we also observed the potential of TXhfq as a naturally delivered vaccine to elicit protective immunity in host against *E. tarda*. Our results provide the first insight to the biological function of *E. tarda* Hfq as well as its applicability in the control of edwardsiellosis in aquaculture.

## Materials and methods

### Ethics statement

Experiments involving live animals were conducted in accordance with the “Regulations for the Administration of Affairs Concerning Experimental Animals” promulgated by the State Science and Technology Commission of Shandong Province. The study was approved by the ethics committee of Institute of Oceanology, Chinese Academy of Sciences. Efforts were taken to ensure that all research animals received good care and humane treatment as stipulated in the above regulations.

### Bacterial strains and growth conditions

*Escherichia coli* BL21(DE3) was purchased from Tiangen (Beijing, China). *E. coli* S17-1λpir was purchased from Biomedal (Sevilla, Spain). *E. tarda* TX01 was isolated from diseased fish [21]. Bacteria were cultured in Luria-Bertani broth (LB) at 37 °C (for *E. coli*) or 28 °C (for *E. tarda*). Where indicated, chloramphenicol, polymyxin B, and 2,2’dipyridyl were supplemented at the concentration of 30 μg/mL, 100 μg/mL, and 100 μM respectively. Biofilm growth on polystyrene surface was conducted as reported previously [22].

### Fish

Clinically healthy Japanese flounder (*Paralichthys olivaceus*) (average 11.9 g) were purchased from a local commercial fish farm. The fish were maintained at ~19 °C in aerated seawater that was changed twice daily. The seawater was sand-filtered and activated carbon-absorbed, with pH of 8.1, oxygen > 6 mg/L, and ammonia < 0.1 mg/L. Fish were acclimatized in the laboratory for two weeks before experimental manipulation. Fish were fed daily with commercial dry pellets (purchased from Shandong Sheng-suo Fish Feed Research Center, Shandong, China) at the amount of ~1.2% body weight. Before experiment, fish (6%) were randomly sampled and examined for the presence of bacteria in blood, liver, kidney, and spleen, and no bacteria were detected from the sampled fish. For tissue collection, fish were euthanized with an overdose of MS222 (tricaine methanesulfonate) (Sigma, USA) as described previously [23].

### Sequence analysis

*hfq* was cloned from *E. tarda* TX01 with primers F1/R1 (Table 1), which were designed based on the known *hfq* sequence of *E. tarda* EIB202 and *E. tarda* FL6-60 (GenBank accession nos. ACY83199.1 and ADM40428.1 respectively). The sequence of Hfq was analyzed using the BLAST program at the National Center for Biotechnology Information (NCBI) and the Expert Protein Analysis System.

**Table 1:** Primers used in this study

**Primer**	**Sequences (5′ → 3′)**^**a**^
F1	ATGGCTAAGGGGCAATCT
R1	TTATTCAGCGTCATCACTGC
F2	*GGATCC*GGATTGATCCGGTCGCC (BamHI)
R2	CTGCCTGCTTGCCCCTTAGCCATTCT
F3	GGCAAGCAGGCAGCAGCTACCA
R3	*GGATCC*CGGTCGGTCTCCAACTG (BamHI)
F4	*GATATC*ATGGCTAAGGGGCAATC (EcoRV)
R4	*GATATC*TTCAGCGTCATCACTGC (EcoRV)
HP1-F	*CCCGGG*ATGCGAGCTGAAACCATTCTG (SmaI)
HP1-R	*CCCGGG*TTTTAAGCATGATGCGCT (SmaI)
HP2-F	*TACGTA*ATGCTGATAATGCAAATATCAACT (SnaBI)
HP2-R	*TACGTA*CAGGTGCGCGTTAAACCACG (SnaBI)
DPFP-F	*GATATC*ATGTCTCAGCCTCAGAGCG (EcoRV)
DPFP-R	*GATATC*CAGCGTCAGCAAACGCGT (EcoRV)

^a^Italicized nucleotides are restriction sites of the enzymes indicated in the brackets at the ends.

### *hfq* knockout

The primers used in this study are listed in Table 1. To construct the *hfq* knockout wild type TXHfq, in-frame deletion of a 222 bp segment (residues 6 to 79) of *hfq* was performed by overlap extension PCR as follows: the first overlap PCR was performed with the primer pair F2/R2, the second overlap PCR was performed with the primer pair F3/R3, and the fusion PCR was performed with the primer pair F2/R3. The PCR products were inserted into the suicide plasmid pDM4 [24] at the BglII site, resulting in pDMHfq. S17-1λpir was transformed with pDMHfq, and the transformants were conjugated with TX01 as described previously [21]. The transconjugants were selected on LB agar plates supplemented with 10% sucrose. One of the colonies that were resistant to sucrose and sensitive to chloramphenicol (marker of pDM4) was analyzed by PCR, and the PCR products were subjected to DNA sequencing to confirm in-frame deletion. This strain was named TXHfq.

### Complementation of *hfq* mutation

The plasmid pJTHfq, which expresses *hfq* constitutively, was created as follows. *hfq* was amplified by PCR with primers F4/R4; the PCR product was ligated with the T-A cloning vector pBS-T (Tiangen, Beijing, China), and the recombinant plasmid was digested with SmaI. The fragment containing *hfq* was retrieved and inserted into plasmid pBT3 [25] at the EcoRV site, resulting in pBT3Hfq. pBT3Hfq was digested with SwaI, and the fragment carrying *hfq* was inserted into plasmid pJT [26] at the SwaI site, resulting in pJTHfq. S17-1λpir was transformed with pJT, and the transformants were conjugated with TXHfq. The transconjugants were selected on LB agar plates supplement with tetracycline (resistance marker of pJT) and polymyxin B. One of the transformants was named TXHfqC.

### H_2_O_2_ survival analysis

H_2_O_2_ survival analysis was performed as reported previously [27].

### Virulence analysis

The median lethal dose (LD_50_) of TX01, TXHfq, and TXHfqC was determined as described previously [23]. For tissue dissemination and colonization analysis, TXHfq and TX01 were cultured in LB medium at 28 °C for different times until OD_600_ of 0.8. The cells were washed with PBS and resuspended in seawater to 1 × 10^8^ CFU/mL. Flounder were immersed in seawater containing TXHfq or TX01 or PBS (control) for 2 h. The fish were then moved to 58-liter tanks containing fresh seawater and reared normally as described above in the section of “Fish”. At 1, 2, 3, 4, 5, and 7 days post-infection (dpi), blood, kidney, liver, and spleen were taken aseptically from the fish (five/time point). The tissues were homogenized in a glass homogenizer containing PBS (100 μL/mg tissue). The homogenates and blood were diluted serially and plated in triplicate on LB agar plates. The plates were incubated at 28 °C for 48 h, and the colonies that appeared on the plates were enumerated. The genetic identity of the colonies was verified by PCR with specific primers [27] and sequence analysis of selected PCR products. The experiment was conducted in three replicates at the same time, and the mean values were given in the results.

### Bacterial replication in macrophages

Flounder head kidney (HK) macrophages were prepared as described previously [28]. The macrophages were cultured in L-15 medium (Thermo Scientific HyClone, Beijing, China) in 96-well culture plates (~10^5^ cells/well). TXHfq, TX01, and TXHfqC suspensions in PBS were prepared as described above and added to macrophages (10^6^ CFU/well). The cells were incubated at 25 °C for 0.5 h. After incubation, the cells were washed with PBS for three times and added with fresh L-15 containing 100 U/mL penicillin and streptomycin (Thermo Scientific HyClone, Beijing, China), followed by incubation at 25 °C for 1.5 h to kill extracellular bacteria. The plates were then washed three times with PBS and incubated at 28 °C for 1 h, 2 h, 4 h, and 8 h. After incubation, the plates were washed with PBS, and the cells were lysed with 100 μL 1% Triton X-100. The cell lysate was serially diluted and plated in triplicate on LB agar plates. The plates were incubated at 28 °C for 48 h, and the colonies that emerged on the plates were counted. The identities of the colonies were verified as described above.

### Reactive oxygen species (ROS) production

ROS production was determined as follows. Flounder HK macrophages in a 96-well microplate (~10^5^ cells/well) were incubated with TXhfq, TX01, or TXhfqC (10^6^ CFU/well) for 1 h or 2 h. The plate was washed with PBS for three times. One hundred microliters of 1 mg/mL nitroblue tetrazolium (Sangon, Shanghai, China) in L-15 was added to the cells. After incubation at 25 °C for 2 h, the reaction was stopped by adding 100% methanol. The plate was washed with 70% methanol, and the reduced formazan was solubilized in 100 μL of 2 M KOH and 120 μL of dimethyl sulfoxide. The plate was read at 630 nm with a microplate reader.

### Nitric oxide (NO) assay

NO production was determined as follows. Flounder HK macrophages in a 96-well microplate (~10^5^ cells/well) were incubated with TXhfq, TX01, or TXhfqC (10^6^ CFU/well) for 1 h or 2 h. The supernatants were removed to a separate 96-well plate (50 μL/well), followed by adding into each well 100 μL of 1% sulphanilamide and 100 μL of 0.1% N-naphthylethylene-diamine (Sigma, St. Louis, MO, USA). The plate was read at 540 nm, and the molar concentration of nitrite was determined from standard curve generated using known concentrations of sodium nitrate.

### Two-dimensional gel electrophoresis (2-DE)

TX01 and TXhfq were cultured in LB medium at 28 °C and collected at OD_600_ 0.8 by centrifugation at 4000 *g* for 15 min at 4 °C. The cells were washed with PBS for three times and resuspended in extraction solution (7 M urea, 2 M thiourea, 4% CHAPS, 40 mM DTT, 2% IPG buffer). The cells were disrupted by intermittent sonic oscillation for a total of 15 min on ice with intervals of 30 s. Unbroken cells and cellular debris were removed by centrifugation at 20 000 *g* for 60 min. The proteins in the supernatant were purified with 2D-Clean-Up Kit (GE Healthcare, Piscataway, NJ, USA) and resuspended in IEF sample loading solution (7 M urea, 2 M thiourea, 2% CHAPS, 40 mM DTT, 0.5% IPG buffer, 0.002% bromophenol blue). Protein concentration was determined using the BCA Protein Assay Kit (Sangon Biotech, Shanghai, China). Two-DE was performed as reported previously [29]. The gel images were acquired using ImageScanner III (GE healthcare, Piscataway, NJ, USA) and analyzed with ImageMaster 2D Platinum 6.0 (GE healthcare, Piscataway, NJ, USA). Triplicate runs were made for each sample to ensure gel reproducibility. For comparative analysis, the percentage intensity volume (%vol) of each spot was used for comparison of matched spots between TXhfq and TX01. To reduce potential errors, a ratio of ≥ 2 (or ≤ 0.5) and analysis of variance (ANOVA) < 0.05 were taken as a threshold for differential expression.

### In-gel enzymatic digestion and matrix-assisted laser desorption/ionization time of flight (MALDI-TOF) mass spectrometry analysis

The differentially expressed protein spots were picked from the gels and washed once with 500 μL water and three times with 500 μL 25 mM ammonium bicarbonate in 50% acetonitrile for 60 min. The gel spots were dehydrated by addition of 500 μL acetonitrile, and the samples were then incubated in 200 μL 10 mM DTT at 56 °C for 60 min to reduce disulfide bonds. Alkylation of cysteines was performed by adding 200 μL 55 mM iodoacetamide, and the samples were incubated at room temperature for 45 min in the dark. The samples were washed with PBS and dehydrated with 500 μL acetonitrile. The samples were incubated in trypsin solution (10 μg/mL in PBS) for 30 min on ice. After incubation, the remaining trypsin solution was removed, and 25 μL of PBS was added to the samples. Proteolysis was performed at 37 °C overnight and stopped by adding 5% formic acid. MALDI-TOF mass spectrometry (MS) analysis was performed with ultrafleXtreme (Bruker, Germany) as follows. One microliter peptide solution was dripped onto the Anchorchip target plate and allowed it to dry at room temperature. Matrix solution (CHCA) was added to the plate, and the plate was loaded into the spectrometer. The mass range was from 500 to 3500 Da, and the scan resolution was 50 000. After the scan, five most abundant MS peaks were selected for MS/MS scan. Protein identification was as described previously [29].

### Quantitative real-time reverse transcription-PCR (qRT-PCR)

TXhfq and TX01 were cultured in in LB medium to an OD_600_ of 0.8. Total RNA was extracted with EZNA Total RNA Kit (Omega Bio-tek, Doraville, GA, USA). The RNA was treated with RNase-free DNaseI (TaKaRa, Dalian, China). One microgram of RNA was used for cDNA synthesis with the Superscript II reverse transcriptase (Invitrogen, Carlsbad, CA, USA). qRT-PCR was carried out in an Eppendorf Mastercycler (Eppendorf, Hamburg, Germany) using SYBR ExScript qRT-PCR Kit (Takara, Dalian, China) as described previously [30]. Melting curve analysis of amplification products was performed at the end of each PCR to confirm that only one PCR product was amplified and detected. The expression level of the target genes was analyzed using comparative threshold cycle method (2^-ΔΔCT^) with 16 s rRNA as an internal control. The data are given in terms of mRNA levels relative to that of 16 s rRNA and expressed as means plus or minus standard errors of the means (SE).

### Antibody preparation and Western blot

To obtain antibodies against hypothetical protein 1 (HP1), hypothetical protein 2 (HP2), and the dyp-type peroxidase family protein (DPFP), His-tagged recombinant proteins of HP1, HP2, and DPFP were prepared. For this purpose, the plasmids pHP1, pHP2, and pDPFP, which express HP1, HP2, and DPFP respectively, were constructed as follows. The coding sequences of HP1, HP2, and DPFP were amplified by PCR with primer pairs HP1-F/HP1-R, HP2-F/HP2-R, and DPFP-F/DPFP-R, respectively (Table 1). The PCR products were ligated with the pEASY-E2 (TransGen, Beijing, China), resulting in pHP1, pHP2, and pDPFP. For protein preparation, *Escherichia coli* BL21(DE3) (Tiangen, Beijing, China) was transformed separately with the plasmids. The transformants were cultured in LB medium at 37 °C to mid-log phase, and expression of recombinant proteins was induced by adding isopropyl-β-D -thiogalactopyranoside to a final concentration of 0.4 mM. Growth was continued at 37 °C for 5 h, and recombinant proteins were purified using Ni-NTA agarose (QIAGEN, Valencia, CA, USA) as recommended by the manufacturer. The purified proteins were dialyzed for 24 h against PBS, and protein concentrations were determined using BCA Protein Assay Kit (Sangon Biotech, Shanghai, China). Rat antibodies against the recombinant proteins were prepared as described previously [31]. For Western blot, equal amounts of proteins from TXhfq and TX01 prepared above were resolved in 12% SDS-PAGE and transferred onto nitrocellulose membranes (Amersham, Cambridge, UK). Immunoblot was performed as reported previously [32] using rat polyclonal antibodies against recombinant HP1, HP2, and DPFP prepared above. Densitometry was performed using the Sensiansy gel analysis system (Shanghai Peiqing Science & Technology. Co., Ltd, China).

### Vaccination

TXhfq was cultured in LB medium as described above and resuspended in seawater to 10^8^ CFU/mL. Japanese flounder were divided into two groups (150 fish/group) named A and B. Group A was immersed in TXhfq bath for 1 h; group B (control) was similarly immersed in seawater containing PBS. After immersion treatment, fish were placed into new 660-liter tanks and washed with fresh seawater to remove any bacteria that had been carried over from the immersion seawater. The fish were then reared under normal conditions as described above in the section of “Fish”. At one month post-vaccination, 50 fish from each group were taken and challenged via intraperitoneal injection with TX01. Similarly, at two months post-vaccination, 50 fish from each group were taken and challenged with TX01. Mortality was monitored over a period of 20 days after challenge. Three dying fish were randomly selected for the examination of bacterial recovery from liver, blood, and spleen as described above. Relative percent of survival (RPS) was calculated according to the following formula: RPS = {1 – (% mortality in vaccinated fish/% mortality in control fish)} × 100. The vaccination experiment was performed in duplicate at the same time.

### Enzyme-linked immunosorbent assay (ELISA)

Sera were taken from vaccinated fish and control fish at one- and two-month post-vaccination and diluted serially in two-fold in PBS. Serum antibody against rEta1 was determined by ELISA analysis as described previously [33]. The assay was conducted with sera from five fish (each as an individual sample), and the mean values were given in the results.

### Statistical analysis

Statistical analyses were performed with the SPSS 18.0 package (SPSS Inc., Chicago, IL, USA). Chi-square test with Yates’ correction was used for mortality analysis, and analysis of variance (ANOVA) was used for all other analyses. Except where otherwise indicated, all in vitro experiments were performed at a single time in three replicates, and the results are shown as means plus or minus standard errors of the means (SE). For in vivo experiments, the number of replicate was indicated in the respective methods. In all cases, significance was defined as *P* < 0.05.

## Results

### Construction of an *E. tarda* Δ*hfq* wild type

Hfq of *E. tarda* is composed of 102 residues and shares 100% and 83.5% overall sequence identities with the Hfq of *Edwardsiella ictaluri* and *E. coli* respectively. To examine its functional importance, the *hfq* gene of *E. tarda* TX01, a highly pathogenic strain, was knocked out by markerless in-frame deletion of a region encoding amino acid residues 6 to 76. The resulting wild type was named TXhfq.

### Mutation of *hfq* has multiple effects

(i.) Effect on growth and survival under different conditions

**Figure 1:**
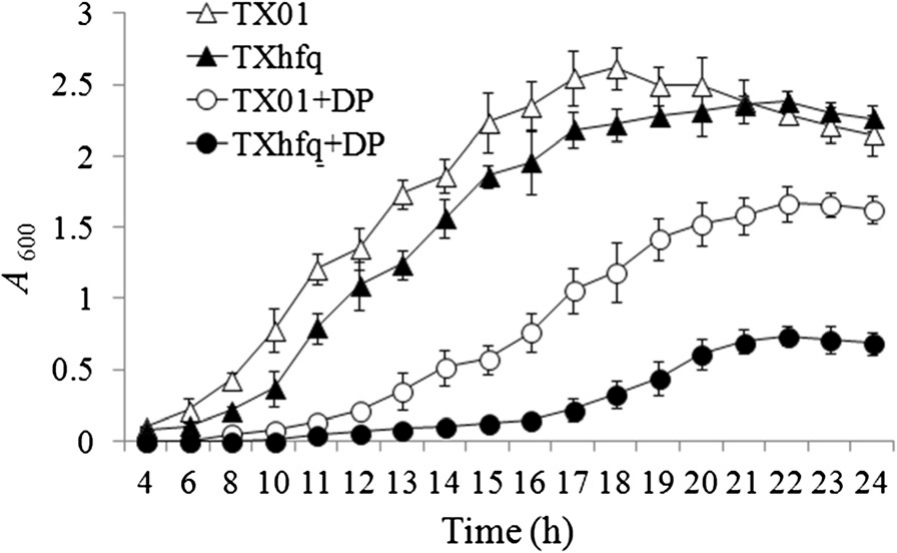
**Growth analysis of TX01 and TXhfq.** TX01 and TXhfq were cultured in LB medium supplemented with or without 2,2′dipyridyl (DP), and cell density was measured at different time points by determining absorbance at OD_600_. Data are presented as means ± SE (N = 3).

Growth analysis showed that when cultured in LB medium, TXhfq exhibited a slower generation time than TX01 at the logarithmic phase but reached similar cell densities as TX01 at the stationary phase (Figure 1). When cultured in LB medium supplemented with the iron chelator 2,2’dipyridyl, the growths of both TX01 and TXhfq were retarded; however, compared to TX01, TXhfq exhibited a much slower growth rate and a much lower maximum cell density at the stationary phase. Examination of biofilm growth on polystyrene surface indicated that TXhfq produced significantly less (3.1 fold) biofilm than TX01 (see Additional file 1). In the presence of H_2_O_2_, which damages bacterial cells via its oxidizing effect, the survival rate of TXhfq (2.8% ± 1.1) was significantly lower than that of TX01 (21.8% ± 2.2).

(ii.) Effect on overall bacterial virulence

**Figure 2:**
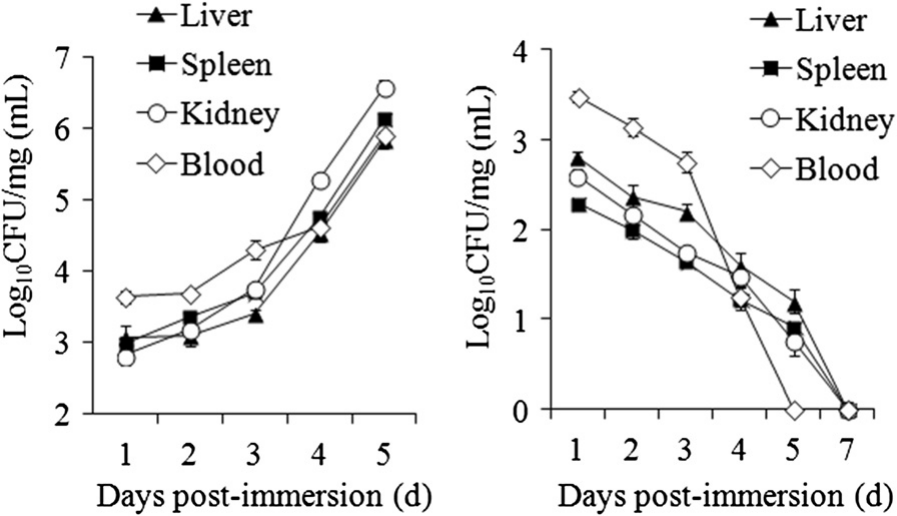
**Invasion of TX01 (A) and TXhfq (B) into fish tissues following infection.** Flounder were infected via immersion with the same dose of TX01 or TXhfq. Bacterial recovery from the liver, spleen, kidney, and blood of the infected fish was determined at different time points (no data on the group of TX01-infected fish at 7 dpi, because no fish survived to this point). The results are the means of three replicates and shown as means ± SE. CFU: colony forming unit. The Y-axis represents the CFU number per mg of tissue (or per mL of blood) expressed in logarithmic form.

Comparative LD_50_ analysis showed that TXhfq exhibited a LD_50_ (1.31 × 10^8^ CFU/fish) that is more than 600 fold higher that of TX01 (1.9 × 10^5^ CFU/fish). When flounder were infected with the same dose of TX01 and TXhfq via immersion, TX01 recoveries from the blood, kidney, liver, and spleen of the infected fish increased from 1, 2, and 3 dpi, and mortality began to occur at 4 dpi (Figure 2A). In contrast, TXhfq recoveries from blood, kidney, liver, and spleen decreased with time and became undetectable after 7 dpi (Figure 2B).

(iii.) Effect on resistance against the immune response of host macrophages

**Figure 3:**
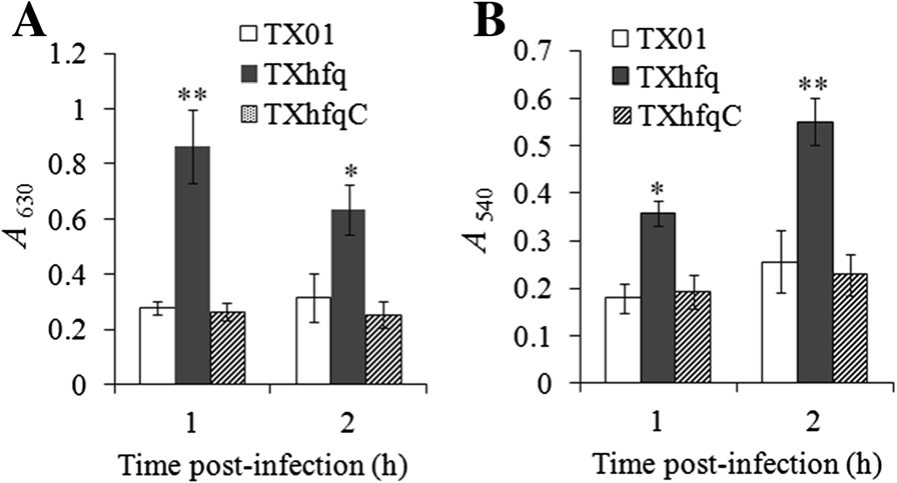
**Effect of TXhfq, TX01, and TXhfqC on the immune response of macrophages.** Flounder head kidney macrophages were infected with TXhfq, TX01, or TXhfqC, and reactive oxygen species **(A)** and nitric oxide **(B)** productions in the cells were determined at 1 h and 2 h post-infection. Data are presented as means ± E (N = 3). *P < 0.05, **P < 0.01.

**Figure 4:**
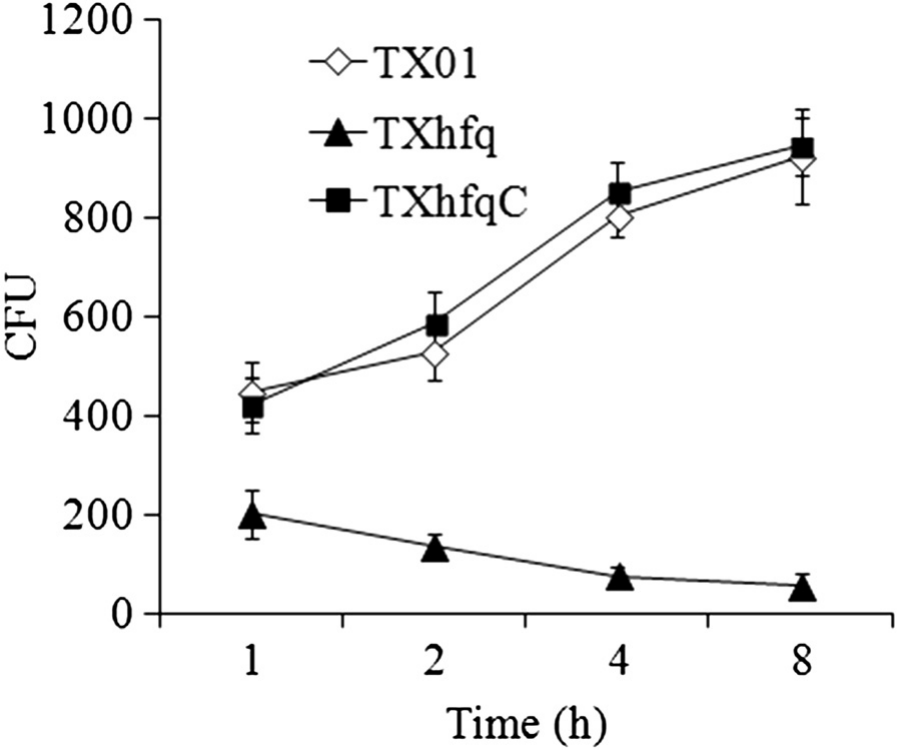
**Multiplication of TXhfq, TX01, and TXhfqC in host macrophages.** Flounder head kidney macrophages were infected with TXhfq, TX01, or TXhfqC. After removing uninfected bacteria, the cells were incubated at 28 °C for different hours, and intracellular bacterial recovery was determined by plate count. Data are presented as means ± E (N = 3).

To examine whether *hfq* mutation affected the ability of *E. tarda* to block activation of host phagocytes, flounder HK macrophages were infected with TXhfq or TX01, and cellular productions of ROS and NO were determined. The results showed that both ROS and NO levels in TXhfq-infected cells were significantly higher than those in TX01-infected cells (Figure 3). Intracellular bacterial recovery analysis showed that after invasion into HK macrophages, TX01 multiplied inside the cells and increased in number as the time progressed, whereas the intracellular number of TXhfq declined with time (Figure 4).

### Genetic complementation of *hfq* deletion and its effect on virulence

To examine whether the virulence defect observed with TXhfq was indeed due to *hfq* deletion, the strain TXhfqC was created, which is a genetic variant of TXhfq that expresses *hfq in trans* from a plasmid. In contrast to TXhfq, TXhfqC exhibited a LD_50_ (1.2 × 10^5^ CFU/fish) comparable to that of TX01. Following infection of flounder HK macrophages, TXhfqC-induced productions of ROS and NO were similar in levels to those induced by TX01 infection (Figure 3). Likewise, the intracellular multiplication capacity of TXhfqC was comparable to that of TX01 (Figure 4).

### Comparative analysis of the protein expression profiles in TXhfq and TX01

(i.) Two-DE protein maps of TXhfq and TX01

**Figure 5:**
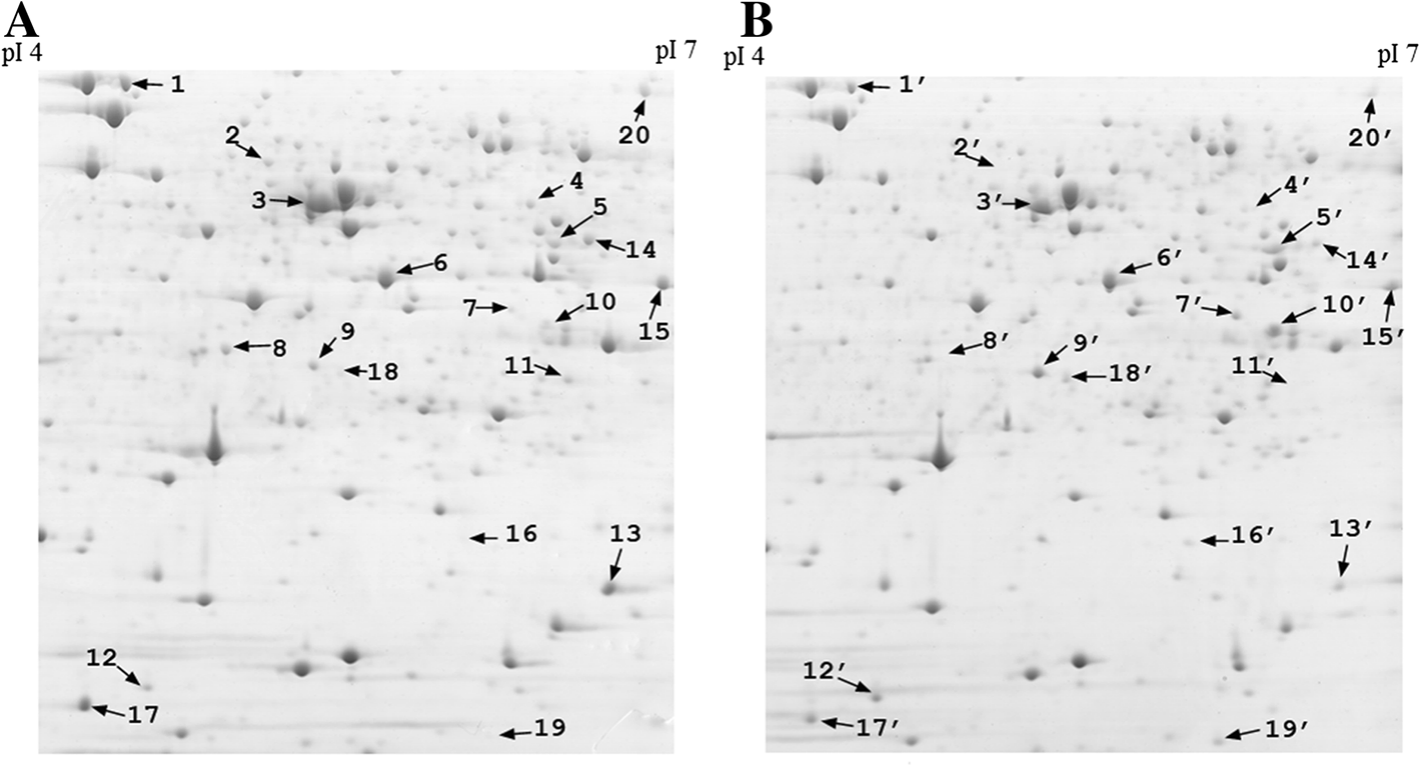
**Representative 2-DE maps showing the protein profiles of TX01 and TXhfq.** Whole cell proteins prepared from TX01 **(A)** and TXhfq **(B)** were subjected to 2-DE analysis. Numbers indicate protein spots with differential expression.

To examine whether there was any difference in the protein profiles of TXhfq and TX01, whole cell proteins of the two strains were subjected to 2-DE analysis. Proteins whose expressions differed by more than 2-fold were further analyzed and listed as putative targets of Hfq regulation. The results showed that 20 protein spots exhibiting apparently differential expressions in TXhfq and TX01 were identified (Figure 5). Of these proteins, eight were significantly upregulated (ratio of TXhfq/TX01 ≥ 2, *P* ≤ 0.05) and twelve were significantly downregulated (ratio of TX01/TXhfq ≥ 2, *P* ≤ 0.05).

(ii.) Mass spectral identification of differentially expressed proteins

The 20 differentially expressed protein spots were subjected to MALDI-TOF/TOF analysis. Fifteen protein spots were successfully identified and grouped into seven functional categories (Table 2). Three proteins (rpsA, Tuf2, and Tsf) belong to the translation group, five proteins (YgeW, ybaS, cysteine synthase, putative iron-containing alcohol dehydrogenase, and dyp-type peroxidase) belong to the metabolism group, one protein (Mrp) is involved in transport, one protein (FkpA) is a putative chaperone, two proteins (OmpR and YqjD) belong to membrane proteins, and one protein (IscU) plays a role in iron-sulfur cluster assembly. The remaining two proteins are hypothetical proteins with unknown functions.

(iii.) Validation of differential expression of selected proteins at mRNA level

**Table 2:** Summary of the differentially expressed proteins

**Spot no.**^**a**^	**NCBI no.**	**Protein name**	**Abrr.**	**MASCOT score**^**b**^	**Theoretical pI/Mr (kD)**	**Coverage (%)**^**c**^	**Fold ratio wild type/wild type (mean ± SD)**^**d**^
**Translation**							
1^e^	gi|269139520	*rpsA* gene product [*Edwardsiella tarda* EIB202]: 30S ribosomal protein S1	RpsA	351	4.61/61.17	41.65	0.42 ± 0.05
3	gi|18858425	*tuf2* gene product [*Edwardsiella tarda* EIB202]: elongation factor Tu	Tuf2	381	4.91/43.43	54.31	0.35 ± 0.09
6	gi|269138094	*tsf* gene product [*Edwardsiella tarda* EIB202]: translation elongation factor Ts	Tsf	223	5.3/30.70	36.84	0.45 ± 0.07
**Metabolism**							
4	gi|294637737	Putative carbamoyltransferase YgeW [*Edwardsiella tarda* ATCC 23685]	YgeW	65.1	6.07/44.38	17.97	0.21 ± 0.12
7	gi|269138485	Dyp-type peroxidase family protein [*Edwardsiella tarda* EIB202]		260	5.76/33.27	38.46	3.62 ± 0.78
8	gi|269140209	*ybaS* gene product [*Edwardsiella tarda* EIB202]: glutaminase	YbaS	362	4.83/33.06	81.35	-∞
14	gi|387866356	Putative iron-containing alcohol dehydrogenase [*Edwardsiella tarda* FL6-60]		218	5.73/42.61	74	0.27 ± 0.06
15	gi|469762796	Cysteine synthase [*Edwardsiella tarda* C07-087]		199	6.03/33.99	77	0.46 ± 0.05
**Transport**							
5	gi|387867244	Mrp (Multiple resistance and pH adaptation) protein [*Edwardsiella tarda* FL6-60]	Mrp	340	6.22/39.70	26.76	2.81 ± 1.11
**Chaperones**							
10	gi|269140581	*fkpA* gene product [*Edwardsiella tarda* EIB202]: FKBP-type peptidyl-prolyl cis-trans isomerase	FkpA	425	8.7/28.76	58.39	4.24 ± 1.35
**Membrane proteins**							
11	gi|269140620|	*ompR* gene product [*Edwardsiella tarda* EIB202]: osmolarity response regulator	OmpR	127	6.31/27.41	38.91	-∞
13	gi|304557842	Uncharacterized membrane protein YqjD [*Edwardsiella tarda* FL6-60]	YqjD	600	6.26/10.94	82.18	0.25 ± 0.04
**Cellular processes**							
12	gi|269140157	*iscU* gene product [*Edwardsiella tarda* EIB202]: FeS cluster assembly scaffold	IscU	102	4.74/13.86	55.47	2.23 ± 0.62
**Unknown function and hypothetical protein**							
2	gi|269139286	Hypothetical protein 1 [*Edwardsiella tarda* EIB202]		153	5.64/58.02	29.04	-∞
9	gi|269140100	Hypothetical protein 2 [*Edwardsiella tarda* EIB202]		318	5.33/33.70	54.90	3.21 ± 1.28
16^e^	NA	NA	NA	NA	NA	NA	5.21 ± 1.25
17	NA	NA	NA	NA	NA	NA	0.31 ± 0.05
18	NA	NA	NA	NA	NA	NA	2.94 ± 0.85
19	NA	NA	NA	NA	NA	NA	4.22 ± 1.77
20	NA	NA	NA	NA	NA	NA	0.32 ± 0.06

^a^Spot ID represents the number on the 2-DE gels (Figure 5).

^b^MOWSE score is -10 log (p), where p is the probability that the observed match is a random event. Based on the NCBInr database using the MASCOT searching program as MS/MS data. Scores greater than 65 are significant (*p* < 0.05).

^c^Number of amino acids spanned by the assigned peptides divided by the protein sequence length.

^d^Mean, the average protein abundance ratio for three paired samples. SD means the standard deviation of protein abundance ratios of one certain spot of three paired samples.

^e^Not analyzed.

**Figure 6:**
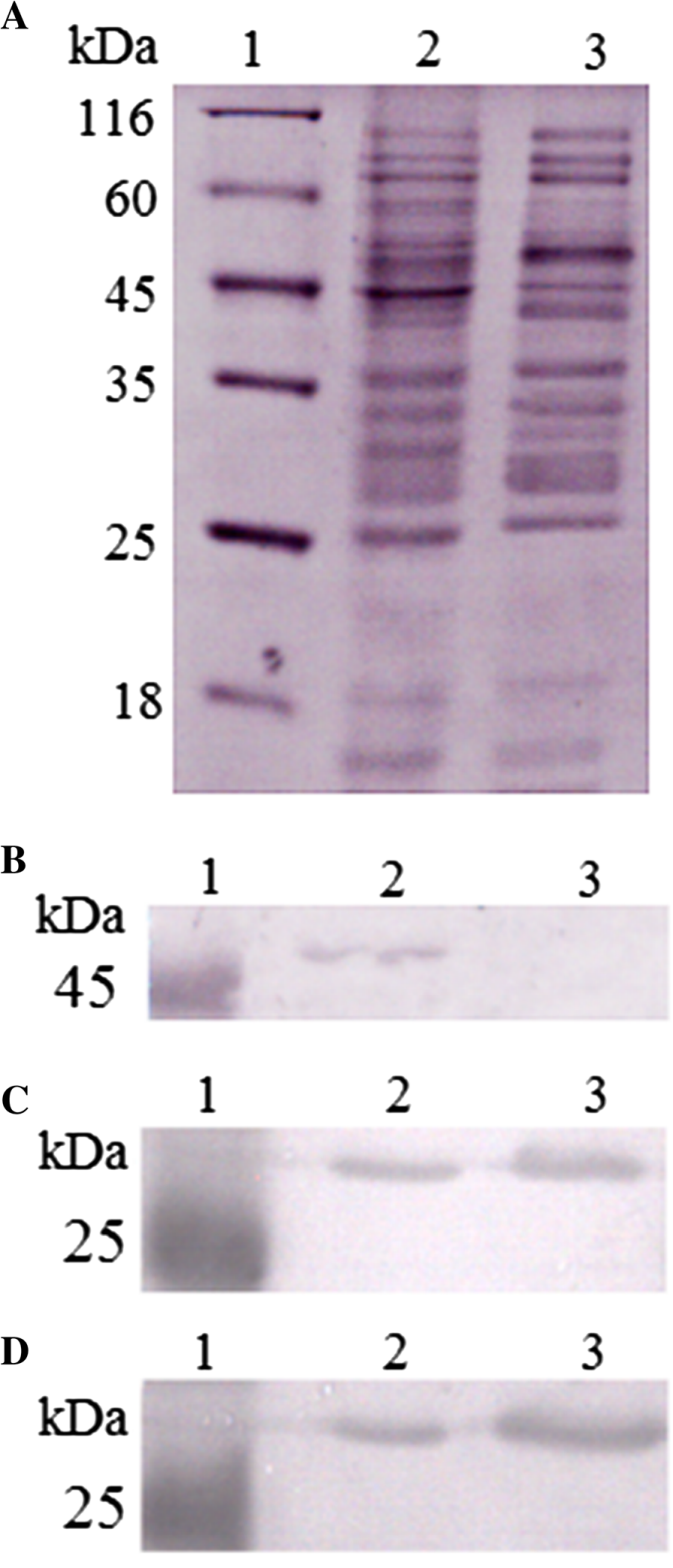
**Immunoblot analysis of differentially expressed proteins.** Equal amounts of proteins prepared from TX01 and TXhfq (lanes 2 and 3 of all panels) were subjected to SDS-PAGE **(A)**. After SDS-PAGE, the proteins were blotted with antibodies against hypothetical protein 1 **(B)**, hypothetical protein 2 **(C)**, or dyp-type peroxidase family protein **(D)**.

qRT-PCR (which determines the mRNA level) (Table 3) showed that the genes encoding Mrp, Dyp-type peroxidase, hypothetical protein 2, and FkpA were upregulated in TXhfq, while the genes encoding hypothetical protein 1, YgeW, ybaS, OmpR, YqjD and cysteine synthase were downregulated in TXhfq, which is consistent with the 2-DE protein profiles. The expressions of the genes encoding RpsA, Tuf2, Tsf, IscU, and the putative iron-containing alcohol dehydrogenase were comparable in the wild type and wild type strains. Three proteins were selected for further analysis by Western blot. The results showed that hypothetical protein 1, which was downregulated in TXhfq by 2-DE and qRT-PCR analysis, was apparently detected in TX01 but was undetectable in TXhfq, while hypothetical protein 2 and the dyp-type peroxidase family protein, both were upregulated in TXhfq by 2-DE and qRT-PCR, were produced more abundantly in TXhfq than in TX01 (fold difference ~2.8 and 4.1 respectively) (Figure 6).

**Table 3:** Summary of mRNA expression in TXhfq (in comparison with that in TX01) as determined by qRT-PCR

**Spo no.**	**Description**	**Protein level (by 2-DE)**	**mRNA level (by qRT-PCR)**
1	*rpsA* gene product [*Edwardsiella tarda* EIB202]: 30S ribosomal protein S1	Down (0.42 ± 0.05)	Unchanged
2	hypothetical protein1 [*Edwardsiella tarda* EIB202]:	Down (-∞)	Down (0.38 ± 0.06)
3	*tuf2* gene product [*Edwardsiella tarda* EIB202]: elongation factor Tu	Down (0.35 ± 0.09)	Unchanged
4	Putative carbamoyltransferase YgeW [*Edwardsiella tarda* ATCC 23685]	Down (0.21 ± 0.12)	Down (0.42 ± 0.09)
5	Mrp(Multiple Resistance and pH adaptation) protein [*Edwardsiella tarda* FL6-60]	Up (2.81 ± 1.11)	Up (4.11 ± 0.85)
6	*tsf* gene product [*Edwardsiella tarda* EIB202]: translation elongation factor Ts	Down (0.45 ± 0.07)	Unchanged
7	Dyp-type peroxidase family protein [*Edwardsiella tarda* EIB202]	Up (3.62 ± 0.78)	Up (7.72 ± 1.08)
8	*ybaS* gene product [*Edwardsiella tarda* EIB202]: glutaminase	Down (-∞)	Down (0.20 ± 0.07)
9	Hypothetical protein [*Edwardsiella tarda* EIB202]: lysophospholipase	Up (3.21 ± 1.28)	Up (5.10 ± 0.84)
10	*fkpA* gene product [*Edwardsiella tarda* EIB202]: FKBP-type peptidyl-prolyl cis-trans isomerase	Up (4.24 ± 1.35)	Up (7.54 ± 1.21)
11	*ompR* gene product [*Edwardsiella tarda* EIB202]: osmolarity response regulator; cytoplasmic	Down (-∞)	Down (0.18 ± 0.04)
12	*iscU* gene product [*Edwardsiella tarda* EIB202]: FeS cluster assembly scaffold	Up (2.23 ± 0.62)	Unchanged
13	Uncharacterized membrane protein YqjD [*Edwardsiella tarda* FL6-60]	Down (0.25 ± 0.04)	Down (0.54 ± 0.05)
14	Putative iron-containing alcohol dehydrogenase [*Edwardsiella tarda* FL6-60]	Down (0.27 ± 0.06)	Unchanged
15	Cysteine synthase [*Edwardsiella tarda* C07-087]	Down (0.46 ± 0.05)	Down (0.33 ± 0.08)

### The potential of TXhfq as an immersion vaccine

Since TXhfq is dramatically decreased in virulence, we examined its potential as a live attenuated vaccine delivered via the natural route. For this purpose, flounder were immunized with live TXhfq via bath immersion. The fish were challenged with TX01 at one month and two months post-vaccination and monitored for mortality. The results showed that mortality began to occur at 4 days and 3 days post-challenge for TXhfq-vaccinated fish and control fish respectively, and that mortality stopped at 14 days and 12 days post-challenge for TXhfq-vaccinated fish and control fish respectively. The accumulated mortalities of TXhfq-vaccinated fish were 24% and 32% at one month and two months post-vaccination respectively, while the accumulated mortalities of the control fish (mock vaccinated with PBS) were 100% and 92% at one month and two months post-vaccination respectively. Based on these results, the protection rates, in terms of PRS, of TXhfq as an immersion vaccine were 76% and 65% respectively at one month and two months post-vaccination. The two protection rates were statistically comparable. Comparable results were obtained in the duplicate vaccination trial, in which the accumulated mortalities of TXhfq-vaccinated fish were 20% and 30% at one month and two months post-vaccination respectively, while the accumulated mortalities of the control fish were 96% and 98% at one month and two months post-vaccination respectively. Examination of moribund fish indicated that TX01 was the only type of bacterium isolated from the liver, spleen, and blood, suggesting that mortality was caused by the experimental challenge. ELISA analysis showed that specific serum antibodies (titers 2^6^ and 2^5^ respectively) were produced in TXhfq-vaccinated fish at one month and two months post-vaccination.

## Discussion

It has been observed that for many bacterial species, mutation of *hfq* has a profound effect on cell growth, while in other bacteria, such as *Haemophilus influenzae* and *Serratia* sp, growth is hardly impaired by *hfq* deletion [19,34,35]. In this study, we found that compared to the wild type TX01, the Δ*hfq* wild type TXhfq exhibited a slight growth difference when cultured in rich medium. However, TXhfq growth was severely retarded when the bacterium was cultured in iron-depleted medium. This result is in agreement with the reports that Hfq plays a critical role in the regulation of iron homeostasis [36-40]. Biofilm growth is a dynamic process with multiple factors involved [41]. Recent studies showed that the major biofilm regulator CsgD was regulated by Hfq-dependent sRNAs in *E. coli* and *Actinobacillus pleuropneumoniae*[42,43], and that in *E. coli*, *Pseudomonas fluorescens*, *Vibrio alginolyticus*, and *Stenotrophomonas maltophilia*, biofilm development was reduced in the absence of Hfq [20,44-46]. Likewise, in our study we found that TXhfq was impaired in the capacity of biofilm production. These results indicate that in *E. tarda*, Hfq is required for both planktonic and biofilm growth. Since for bacterial pathogens, biofilm production is often associated with infectivity, these observations suggest that Hfq possibly plays a role under both physiological conditions and during bacterial infection.

Reports have shown that Hfq is essential to cellular tolerance of various stresses, such as oxidative damage, high salt, and heat shock, in a variety of bacteria including *Serratia* sp, *E. coli*, *Francisella tularensis*, *K. pneumoniae*, and *V. alginolyticus*[19,47]. However, in other bacteria such as *Listeria monocytogenes*, *Haemophilus influenzae*, and *Staphylococcus aureus*, Hfq has no effect on resistance to oxidative stress [34,48]. In the case of *E. tarda*, we found that in the presence of the strong oxidizer H_2_O_2_, the survival rate of TXhfq was significantly lower than that of TX01, suggesting that Hfq is required for coping with oxidative stress in *E. tarda*. Given the fact that production of reactive oxygen species, which induces a state of oxidative stress, is a key defense mechanism in fish as well as mammals against bacterial pathogens, these results indicate a potential involvement of Hfq in the virulence *E. tarda*.

A large amount of evidences have indicated that Hfq plays a role in pathogenicity. For example, mutation of *hfq* affects the ability of *Brucella melitensis* and *Salmonella enterica* serovar Typhimurium to invade and proliferate inside host cells [49,50]. Similar to these observations, we found that TXhfq exhibited a dramatically increased LD_50_, and, consistently, the ability of TXhfq to disseminate in flounder tissues and replicate in HK macrophages was significantly impaired. In addition, we found that HK macrophages infected with TXhfq produced much higher levels of ROS and NO than macrophages infected with TX01, suggesting that TXhfq was defective in blocking macrophage activation. In agreement with these results, TXhfq exhibited reduced capacity to survive and replicate inside HK macrophages. These results indicate that Hfq is essential for effective repression of the bactericidal immune response of host cells. Previous studies have shown that *E. tarda* is an intracellular pathogen that can escape host immune defense and replicate in host phagocytes [51-53]; however, the mechanism behind this phenomenon is unclear. Our results confirm previous observations and suggest a close link between immune evasion and *E. tarda* virulence. More importantly, our results, together with those of previous reports, point to a possibility that the intracellular replication capacity of *E. tarda* is not due to the action of a single bacterial factor but more likely to the combined effects of multiple factors.

Hfq is known to regulate gene expression and mRNA stability in post-transcription level, resulting in changes in protein production. Hence, proteomics is an appropriate strategy to detect putative targets of Hfq. Using this approach, Hfq regulons have been identified in *Salmonella*, *Sinorhizobium meliloti,* and *Neisseria meningitides*[39,54-57]. In this study, global proteomic analysis identified 15 proteins differentially expressed between TXhfq and TX01. Of these proteins, YgeW is a carbamoyltransferase that is likely involved in the biosynthesis of antibiotic [58], YbaS is a glutaminase known to contribute to acid resistance [59,60], iron-containing alcohol dehydrogenase and cysteine synthase have been reported to participate in regulation of ethanol utilization and production of antioxidant respectively [61,62], YqjD is an inner membrane protein associated with stationary-phase ribosomes [63], and OmpR is an outer membrane protein that is essential for low pH adaptation and regulates the virulence-associated type VI secretion system [64]. The downregulated expression of these proteins observed in TXhfq may account in part for the defectiveness of TXhfq in growth and in coping with stress conditions. qRT-PCR analysis of the genes encoding the 15 differentially expressed proteins showed that for ten genes, the mRNA levels were consistent with the 2-DE results, while for five genes, the mRNA levels were comparable between TXhfq and TX01. These results suggest that in most cases Hfq regulates the target genes at the transcription level, while in some cases Hfq regulates the target genes at the posttranscriptional level. It will be interesting for future studies to delineate the detail process of Hfq regulation of different types of targets. In addition, since none of the differentially expressed proteins has been studied in *E. tarda*, works may be carried out in the future to inactivate these proteins (e.g. by gene knock-out) and investigate the potential significance of these proteins in *E. tarda* survival under different conditions.

Since Hfq wild types usually exhibit vitiated virulence, they are ideal targets for the development of attenuated live vaccines. This idea has been exploited by researchers with different pathogenic species such as *S. enterica* serovar Typhimurium, *Brucella melitensis*, and *V. alginolyticus*[45,65,66]. In our study, we found that flounder immunized with live TXhfq via bath immersion exhibited high levels of survival rates at one- and two-month post-vaccination after lethal *E. tarda* challenge, suggesting that TXhfq confers effective protection against *E. tarda*. Given the fact that TXhfq, though highly attenuated in virulence, is still capable of transient infection into flounder via immersion as observed in the tissue dissemination analysis, it is likely that TXhfq mimics natural infection after immersion vaccination and thus induces strong protective immunity in the host. The advantage of TXhfq as a vaccine lies not only in its protectivity but also in its immersion delivery, which for fish is a natural approach. Compared to the commonly used injection method of vaccine delivery, immersion is of low cost and inflicts no stress upon the animals.

In conclusion, we demonstrate in this study that *hfq* knockout affects multiple aspects of *E. tarda*, which results in dramatic attenuation of infectivity. Hfq is required for the expression of a wide range of proteins belonging to different functional categories, and the regulatory effects of Hfq likely exert at both transcription and post-transcription levels. In addition, the Δ*hfq* wild type as an immersion vaccine induces effective immunoprotection, a property that may be exploited for the control of *E. tarda* in aquaculture.

## Competing interests

The authors declare that they have no competing interests.

## Authors’ contributions

YHH and YXL performed the experiments and analyzed the data. LS designed the experiment. LS and YHH wrote the paper. All authors read and approved the final manuscript.

## Supplementary Material

Additional file 1 **Biofilm production of TX01 and TXhfq.** TX01 and TXhfq were grown in polystyrene plates for 24 h and then assayed for biofilm production. Data are presented as means ± E (N = 3). **, *P* < 0.01. Click here for file

## References

[B1] Mohanty BR Sahoo PK Edwardsiellosis in fish: a brief review J Biosci 2007 32 1331 1344 10.1007/s12038-007-0143-8 18202458

[B2] Park SB Aoki T Jung TS Pathogenesis of and strategies for preventing *Edwardsiella tarda* infection in fish Vet Res 2012 43 67 10.1186/1297-9716-43-67 23035843 PMC3479428

[B3] Nelson JJ Nelson CA Carter JE Extraintestinal manifestations of *Edwardsiella tarda* infection: a 10-year retrospective review J La State Med Soc 2009 161 103 106 19489391

[B4] Leung KY Siame BA Tenkink BJ Noort RJ Mok YK *Edwardsiella tarda*–virulence mechanisms of an emerging gastroenteritis pathogen Microbes Infect 2012 14 26 34 10.1016/j.micinf.2011.08.005 21924375

[B5] de Fernandez MT F Eoyang L August JT de Fernandez MT F Eoyang L August JT Factor fraction required for the synthesis of bacteriophage QBeta-RNA Nature 1968 219 588 590 10.1038/219588a0 4874917

[B6] De Lay N Schu DJ Gottesman S Bacterial small RNA-based negative regulation: Hfq and its accomplices J Biol Chem 2013 288 7996 8003 10.1074/jbc.R112.441386 23362267 PMC3605619

[B7] Vogel J Luisi BF Hfq and its constellation of RNA Nat Rev Microbiol 2011 9 578 589 10.1038/nrmicro2615 21760622 PMC4615618

[B8] Bibova I Skopova K Masin J Cerny O Hot D Sebo P Vecerek B The RNA chaperone Hfq is required for virulence of *Bordetella pertussis* Infect Immun 2013 81 4081 4090 10.1128/IAI.00345-13 23980112 PMC3811842

[B9] Bojer MS Jakobsen H Struve C Krogfelt KA Løbner-Olesen A Lack of the RNA chaperone Hfq attenuates pathogenicity of several *Escherichia coli* pathotypes towards *Caenorhabditis elegans* Microbes Infect 2012 14 1034 1309 10.1016/j.micinf.2012.06.002 22713744

[B10] Caswell CC Gaines JM Roop RM 2nd The RNA chaperone Hfq independently coordinates expression of the VirB type IV secretion system and the LuxR-type regulator BabR in *Brucella abortus* 2308 J Bacteriol 2012 194 3 14 10.1128/JB.05623-11 22020650 PMC3256608

[B11] Chiang MK Lu MC Liu LC Lin CT Lai YC Impact of Hfq on global gene expression and virulence in *Klebsiella pneumoniae* PLoS One 2011 6 e22248 10.1371/journal.pone.0022248 21779404 PMC3136514

[B12] Ding Y Davis BM Waldor MK Hfq is essencial for *Vibrio cholera* virulence and dowregulates sigma expression Mol Microbiol 2004 53 345 354 10.1111/j.1365-2958.2004.04142.x 15225327

[B13] Meng X Meng X Zhu C Wang H Wang J Nie J Hardwidge PR Zhu G The RNA chaperone Hfq regulates expression of fimbrial-related genes and virulence of *Salmonella enterica* serovar Enteritidis FEMS Microbiol Lett 2013 346 90 96 10.1111/1574-6968.12206 23808344

[B14] Nakao H Watanabe H Nakayama S Takeda T *yst* gene expression in *Yersinia enterocolitica* is positively regulated by a chromosomal region that is highly homologous to *Escherichia coli* host factor 1 gene (*hfq*) Mol Microbiol 2005 18 859 865 8825090 10.1111/j.1365-2958.1995.18050859.x

[B15] Sittka A Pfeiffer V Tedin K Vogel J The RNA chaperone Hfq is essential for the virulence of *Salmonella typhimurium* Mol Microbiol 2007 63 193 217 10.1111/j.1365-2958.2006.05489.x 17163975 PMC1810395

[B16] Sonnleitner E Hagens S Rosenau F Wilhelm S Habel A Jäger KE Bläsi U Reduced virulence of a *hfq* wild type of *Pseudomonas aeruginosa* O1 Microb Pathol 2003 35 217 228 10.1016/S0882-4010(03)00149-9 14521880

[B17] Wilms I Möller P Stock AM Gurski R Lai EM Narberhaus F Hfq influences multiple transport systems and virulence in the plant pathogen *Agrobacterium tumefaciens* J Bacteriol 2012 194 5209 5217 10.1128/JB.00510-12 22821981 PMC3457239

[B18] Zeng Q McNally RR Sundin GW Global small RNA chaperone Hfq and regulatory small RNAs are important virulence regulators in *Erwinia amylovora* J Bacteriol 2013 195 1706 1717 10.1128/JB.02056-12 23378513 PMC3624556

[B19] Chao Y Vogel J The role of Hfq in bacterial pathogens Curr Opin Microbiol 2010 13 24 33 10.1016/j.mib.2010.01.001 20080057

[B20] Roscetto E Angrisano T Costa V Casalino M Förstner KU Sharma CM Di Nocera PP De Gregorio E Functional characterization of the RNA chaperone Hfq in the opportunistic human pathogen *Stenotrophomonas maltophilia* J Bacteriol 2012 194 5864 5874 10.1128/JB.00746-12 22923593 PMC3486118

[B21] Sun K Wang HL Zhang M Xiao ZZ Sun L Genetic mechanisms multi-antimicrobial resistance in a pathogenic *Edwardsiella tarda* strain Aquaculture 2009 289 134 139 10.1016/j.aquaculture.2008.12.021

[B22] Hu YH Liu CS Hou JH Sun L Identification, characterization, and molecular application of a virulence-associated autotransporter from a pathogenic *Pseudomonas fluorescens* strain Appl Environ Microbiol 2009 75 4333 4340 10.1128/AEM.00159-09 19447960 PMC2704808

[B23] Wang HR Hu YH Zhang WW Sun L Construction of an attenuated *Pseudomonas fluorescens* strain and evaluation of its potential as a cross-protective vaccine Vaccine 2009 27 4047 4055 10.1016/j.vaccine.2009.04.023 19501788

[B24] Milton DL O’Toole R Horstedt P Wolf-Watz H Flagellin A is essential for the virulence of *Vibrio anguillarum* J Bacteriol 1996 178 1310 1319 8631707 10.1128/jb.178.5.1310-1319.1996 PMC177804

[B25] Zhang WW Sun K Cheng S Sun L Characterization of DegQVh, a serine protease and a protective immunogen from a pathogenic *Vibrio harveyi* strain Appl Environ Microbiol 2008 74 6254 6262 10.1128/AEM.00109-08 18723647 PMC2570273

[B26] Hu YH Wang HL Zhang M Sun L Molecular analysis of the copper-responsive CopRSCD of a pathogenic *Pseudomonas fluorescens* strain J Microbiol 2009 47 277 286 10.1007/s12275-008-0278-9 19557345

[B27] Zheng WJ Hu YH Sun L The two Dps of *Edwardsiella tarda* are involved in resistance against oxidative stress and host infection Fish Shellfish Immunol 2011 31 985 992 21907291 10.1016/j.fsi.2011.08.018

[B28] Liu CS Sun Y Hu YH Sun L Identification and analysis of the immune effects of CpG motifs that protect Japanese flounder (*Paralichthys olivaceus*) against bacterial infection Fish Shellfish Immunol 2010 29 279 285 10.1016/j.fsi.2010.04.012 20420914

[B29] Zhang J Hu YH Xiao ZZ Sun L Megalocytivirus-induced proteins of turbot (*Scophthalmus maximus*): identification and antiviral potential J Proteomics 2013 91 430 443 23933595 10.1016/j.jprot.2013.07.033

[B30] Zheng WJ Sun L Evaluation of housekeeping genes as references for quantitative real time RT-PCR analysis of gene expression in Japanese flounder (*Paralichthys olivaceus*) Fish Shellfish Immunol 2011 30 638 645 10.1016/j.fsi.2010.12.014 21185941

[B31] Liu CS Sun Y Zhang M Sun L Identification and analysis of a *Sciaenops ocellatus* ISG15 homologue that is involved in host immune defense against bacterial infection Fish Shellfish Immunol 2010 29 167 174 10.1016/j.fsi.2010.03.012 20385242

[B32] Sun Y Zheng WJ Hu YH Sun BG Sun L *Edwardsiella tarda* Eta1, an in vivo-induced antigen that is involved in host infection Infect Immun 2012 80 2948 2955 10.1128/IAI.00063-12 22585967 PMC3434574

[B33] Sun Y Liu CS Sun L Identification of an *Edwardsiella tarda* surface antigen and analysis of its immunoprotective potential as a purified recombinant subunit vaccine and a surface-anchored subunit vaccine expressed by a fish commensal strain Vaccine 2010 28 6603 6608 10.1016/j.vaccine.2010.07.050 20673823

[B34] Hempel RJ Morton DJ Seale TW Whitby PW Stull TL The role of the RNA chaperone Hfq in *Haemophilus influenzae* pathogenesis BMC Microbiol 2013 13 134 10.1186/1471-2180-13-134 23767779 PMC3691723

[B35] Wilf NM Williamson NR Ramsay JP Poulter S Bandyra KJ Salmond GP The RNA chaperone, Hfq, controls two luxR-type regulators and plays a key role in pathogenesis and production of antibiotics in *Serratia* sp. ATCC 39006 Environ Microbiol 2011 13 2649 2666 10.1111/j.1462-2920.2011.02532.x 21824244

[B36] Bearson BL Bearson SM Uthe JJ Dowd SE Houghton JO Lee I Toscano MJ Lay DC Jr Iron regulated genes of *Salmonella enterica* serovar Typhimurium in response to norepinephrine and the requirement of fepDGC for norepinephrine-enhanced growth Microbes Infect 2008 10 807 816 10.1016/j.micinf.2008.04.011 18554972

[B37] Mellin JR McClure R Lopez D Green O Reinhard B Genco C Role of Hfq in iron-dependent and -independent gene regulation in *Neisseria meningitidis* Microbiology 2010 56 2316 2326 20430815 10.1099/mic.0.039040-0 PMC3068672

[B38] Metruccio MM Fantappiè L Serruto D Muzzi A Roncarati D Donati C Scarlato V Delany I The Hfq-dependent small noncoding RNA NrrF directly mediates Fur-dependent positive regulation of succinate dehydrogenase in *Neisseria meningitidis* J Bacteriol 2009 191 1330 1342 10.1128/JB.00849-08 19060140 PMC2631994

[B39] Sobrero P Schlüter JP Lanner U Schlosser A Becker A Valverde C Quantitative proteomic analysis of the Hfq-regulon in *Sinorhizobium meliloti* 2011 PLoS One 2012 7 e48494 10.1371/journal.pone.0048494 23119037 PMC3484140

[B40] Vecerek B Moll I Afonyushkin T Kaberdin V Blasi U Interaction of the RNA chaperone Hfq with mRNAs: direct and indirect roles of Hfq in iron metabolism of *Escherichia coli* Mol Microbiol 2003 50 897 909 10.1046/j.1365-2958.2003.03727.x 14617150

[B41] Salazar JK Wu Z Yang W Freitag NE Tortorello ML Wang H Zhang W Roles of a novel Crp/Fnr family transcription factor Lmo0753 in soil survival, biofilm production and surface attachment to fresh produce of *Listeria monocytogenes* PLoS One 2013 8 e75736 10.1371/journal.pone.0075736 24066185 PMC3774658

[B42] Mika F Busse S Possling A Berkholz J Tschowri N Sommerfeldt N Pruteanu M Hengge R Targeting of csgD by the small regulatory RNA RprA links stationary phase, biofilm formation and cell envelope stress in *Escherichia coli* Mol Microbiol 2012 84 51 65 10.1111/j.1365-2958.2012.08002.x 22356413 PMC3465796

[B43] Subashchandrabose S Leveque RM Kirkwood RN Kiupel M Mulks MH The RNA chaperone Hfq promotes fitness of *Actinobacillus pleuropneumoniae* during porcine pleuropneumonia Infect Immun 2013 81 2952 2961 10.1128/IAI.00392-13 23732171 PMC3719588

[B44] Kulesus RR Diaz-Perez K Slechta ES Eto DS Mulvey MA Impact of the RNA chaperone Hfq on the fitness and virulence potential of uropathogenic *Escherichia coli* Infect Immun 2008 76 3019 3026 10.1128/IAI.00022-08 18458066 PMC2446724

[B45] Liu H Wang Q Liu Q Cao X Shi C Zhang Y Roles of Hfq in the stress adaptation and virulence in fish pathogen *Vibrio alginolyticus* and its potential application as a target for live attenuated vaccine Appl Microbiol Biotechnol 2011 91 353 364 10.1007/s00253-011-3286-3 21523476

[B46] Wu XG Duan HM Tian T Yao N Zhou HY Zhang LQ Effect of the hfq gene on 2,4-diacetylphloroglucinol production and the PcoI/PcoR quorum-sensing system in *Pseudomonas fluorescens* 2P24 FEMS Microbiol Lett 2010 309 16 24 20528945 10.1111/j.1574-6968.2010.02009.x

[B47] Christiansen JK Larsen MH Ingmer H Sogaard-Andersen L Kallipolitis BH The RNA-binding protein Hfq of *Listeria monocytogenes*: role in stress tolerance and virulence J Bacteriol 2004 186 3355 3362 10.1128/JB.186.11.3355-3362.2004 15150220 PMC415768

[B48] Bohn C Rigoulay C Bouloc P No detectable effect of RNA-binding protein Hfq absence in *Staphylococcus aureus* BMC Microbiol 2007 7 10 10.1186/1471-2180-7-10 17291347 PMC1800855

[B49] Cui M Wang T Xu J Ke Y Du X Yuan X Wang Z Gong C Zhuang Y Lei S Su X Wang X Huang L Zhong Z Peng G Yuan J Chen Z Wang Y Impact of Hfq on global gene expression and intracellular survival in *Brucella melitensis* PLoS One 2013 8 e71933 10.1371/journal.pone.0071933 23977181 PMC3747064

[B50] Viegas SC Mil-Homens D Fialho AM Arraiano CM The Virulence of *Salmonella enterica* Serovar Typhimurium in the insect model galleria mellonella is impaired by mutations in RNase E and RNase III Appl Environ Microbiol 2013 79 6124 6133 10.1128/AEM.02044-13 23913419 PMC3811362

[B51] Cheng S Zhang M Sun L The iron-cofactored superoxide dismutase of *Edwardsiella tarda* inhibits macrophage-mediated innate immune response Fish Shellfish Immunol 2010 29 972 978 10.1016/j.fsi.2010.08.004 20732430

[B52] Ishibe K Osatomi K Hara K Kanai K Yamaguchi K Oda T Comparison of the responses of peritoneal macrophages from Japanese flounder (*Paralichthys olivaceus*) against high virulent and low virulent strains of *Edwardsiella tarda* Fish Shellfish Immunol 2008 24 243 251 10.1016/j.fsi.2007.11.001 18178102

[B53] Rao PSS Lim TM Leung KY Opsonized virulent *Edwardsiella tarda* strains are able to adhere to and survive and replicate within fish phagocytes but fail to stimulate reactive oxygen intermediates Infect Immun 2001 69 5689 5697 10.1128/IAI.69.9.5689-5697.2001 11500445 PMC98685

[B54] Ansong C Yoon H Porwollik S Mottaz-Brewer H Petritis BO Jaitly N Adkins JN McClelland M Heffron F Smith RD Global systems-level analysis of Hfq and SmpB deletion wild types in Salmonella: implications for virulence and global protein translation PLoS One 2009 4 e4809 10.1371/journal.pone.0004809 19277208 PMC2652828

[B55] Barra-Bily L Fontenelle C Jan G Flechard M Trautwetter A Pandey SP Walker GC Blanco C Proteomic alterations explain phenotypic changes in *Sinorhizobium meliloti* lacking the RNA chaperone Hfq J Bacteriol 2010 192 1719 1729 10.1128/JB.01429-09 20081032 PMC2832530

[B56] Fantappie L Metruccio MM Seib KL Oriente F Cartocci E Ferlicca F Giuliani MM Scarlato V Delany I The RNA chaperone Hfq is involved in the stress response and virulence in *Neisseria meningitidis* and is a pleiotropic regulator of protein expression Infect Immun 2009 77 1842 1853 10.1128/IAI.01216-08 19223479 PMC2681778

[B57] Pannekoek Y Huis in’t Veld R Hopman CT Langerak AA Speijer D van der Ende A Molecular characterization and identification of proteins regulated by Hfq in *Neisseria meningitidis* FEMS Microbiol Lett 2009 294 216 224 10.1111/j.1574-6968.2009.01568.x 19374669 PMC2734931

[B58] Hong YS Lee D Kim W Jeong JK Kim CG Sohng JK Lee JH Paik SG Lee JJ Inactivation of the carbamoyltransferase gene refines post-polyketide synthase modification steps in the biosynthesis of the antitumor agent geldanamycin J Am Chem Soc 2004 126 11142 11143 10.1021/ja047769m 15355082

[B59] Brown G Singer A Proudfoot M Skarina T Kim Y Chang C Dementieva I Kuznetsova E Gonzalez CF Joachimiak A Savchenko A Yakunin AF Functional and structural characterization of four glutaminases from *Escherichia coli* and *Bacillus subtilis* Biochemistry 2008 47 5724 5735 10.1021/bi800097h 18459799 PMC2735108

[B60] Lu P Ma D Chen Y Guo Y Chen GQ Deng H Shi Y L-glutamine provides acid resistance for *Escherichia coli* through enzymatic release of ammonia Cell Res 2013 23 635 644 10.1038/cr.2013.13 23337585 PMC3641589

[B61] Hempel N Görisch H Mern DS Gene ercA encoding a putative iron-containing alcohol dehydrogenase is involved in regulation of ethanol utilization in *Pseudomonas aeruginosa* J Bacteriol 2013 195 3925 3932 10.1128/JB.00531-13 23813731 PMC3754586

[B62] O’Leary SE Jurgenson CT Ealick SE Begley TP O-phospho-L-serine and the thiocarboxylated sulfur carrier protein CysO-COSH are substrates for CysM, a cysteine synthase from *Mycobacterium tuberculosis* Biochemistry 2008 47 11606 11615 10.1021/bi8013664 18842002 PMC2647513

[B63] Yoshida H Maki Y Furuike S Sakai A Ueta M Wada A YqjD is an inner membrane protein associated with stationary-phase ribosomes in *Escherichia coli* J Bacteriol 2012 194 4178 4183 10.1128/JB.00396-12 22661687 PMC3416271

[B64] Zhang W Wang Y Song Y Wang T Xu S Peng Z Lin X Zhang L Shen X A type VI secretion system regulated by OmpR in *Yersinia pseudotuberculosis* functions to maintain intracellular pH homeostasis Environ Microbiol 2013 15 557 569 10.1111/1462-2920.12005 23094603

[B65] Allam US Krishna MG Lahiri A Joy O Chakravortty D *Salmonella enterica* serovar Typhimurium lacking hfq gene confers protective immunity against murine typhoid PLoS One 2011 6 e16667 10.1371/journal.pone.0016667 21347426 PMC3036662

[B66] Zhang J Guo F Chen C Li Z Zhang H Wang Y Zhang K Du G Li Y Wang J Jian T Wang Z *Brucella melitensis* 16MΔhfq attenuation confers protection against wild-type challenge in BALB/c mice Microbiol Immunol 2013 57 502 510 23647412 10.1111/1348-0421.12065

