# Supplementary figures and images for: Edwardsiella tarda Hfq: impact on host infection and global protein expression
Source: Vet Res. 2014 Feb 25;45(1):23. doi: 10.1186/1297-9716-45-23 (PMC4015145; doi:10.1186/1297-9716-45-23)

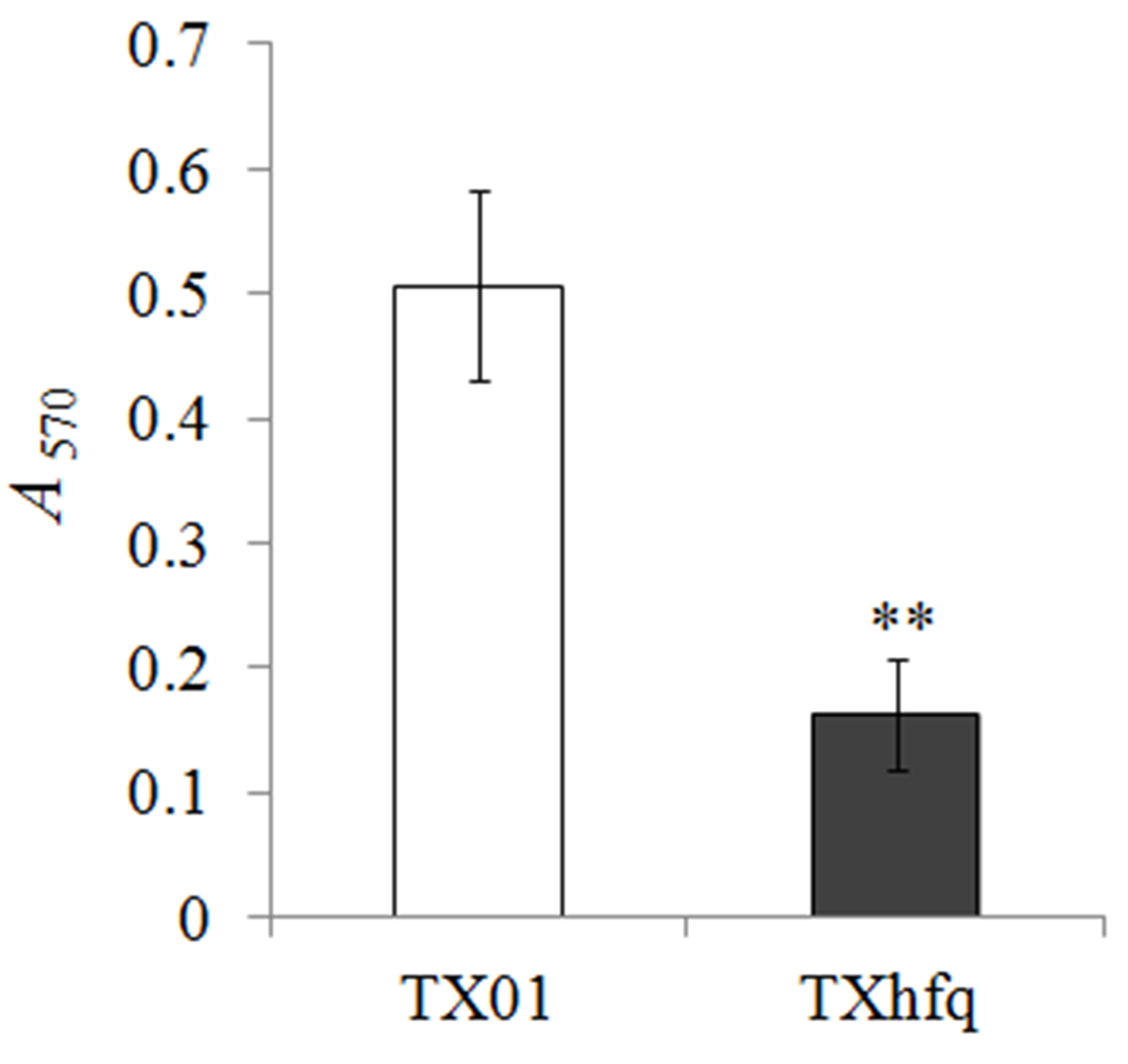

Supplement: Additional file 1 — Biofilm production of TX01 and TXhfq. TX01 and TXhfq were grown in polystyrene plates for 24 h and then assayed for biofilm production. Data are presented as means ± E (N = 3). **, P < 0.01. [file 1297-9716-45-23-S1.tiff]
